# Supplementary material for: Redefining the specificity of phosphoinositide-binding by human PH domain-containing proteins
Source: Nat Commun. 2021 Jul 15;12:4339. doi: 10.1038/s41467-021-24639-y (PMC8282632; doi:10.1038/s41467-021-24639-y)
Supplement: Supplementary file 1 — Supplementary Information [file 41467_2021_24639_MOESM1_ESM.pdf]

Supplementary Information for:

Redefining the specificity of phosphoinositide-binding by human PH domain-containing proteins

Nilmani Singh[1†], Adriana Reyes-Ordoñez[1†], Michael A. Compagnone[1], Jesus F. Moreno[1], Benjamin J. Leslie[2], Taekjip Ha[2,3,4,5], Jie Chen[1*]

[1]Department of Cell & Developmental Biology, University of Illinois at Urbana-Champaign, Urbana, IL 61801; [2]Department of Biophysics and Biophysical Chemistry, Johns Hopkins University School of Medicine, Baltimore, MD 21205; [3]Department of Biophysics, Johns Hopkins University, Baltimore, MD 21218; [4]Department of Biomedical Engineering, Johns Hopkins University, Baltimore, MD 21205; [5]Howard Hughes Medical Institute, Baltimore, MD 21205, USA

[†]These authors contributed equally to this work.
[*]Correspondence: jiechen@illinois.edu.

PH Domain	Lipid binding by PH domain	Lipid binding by full-length protein
ACAP1_PH	PI(3,4)P_2	PI(3,4)P_2, PI(4)P
ADAP1_PH1	None	PI(3,4,5)P_3, PI(4,5)P_2, PI(3,4)P_2
ADAP1_PH2	None	PI(3,4,5)P_3, PI(4,5)P_2, PI(3,4)P_2
ARHGEF3-PH	Various PIPs, see Figure 3	PI(4,5)P_2, PI(3,5)P_2
ARHGEF5_PH	None	PI(4,5)P_2, PI(3,4)P_2
ARHGEF9_PH	None	PI(5)P
DOK2-PH	PI(3,4,5)P3, PI(3)P, PI(4)P	PI(3)P
FERMT3-PH	None	PI(3,4,5)P_3, PI(3,4)P_2
PLEKHA1-PH1	None	PI(3,4)P_2
PLEKHA1-PH2	PI(3,4)P_2	PI(3,4)P_2
SPATA13-PH	None	Bound all lipid vesicles
VAV1-PH	None	PI(3,4,5)P_3, PI(4,5)P_2, PI(3,4)P_2, PI(3,5)P_2, PI(5)P

Supplementary Table 1. SiMPull assay results for selected PH domains. All fusion proteins had EGFP at the N-terminus except that ARHGEF3-PH was analyzed with both N-terminus and C-terminus fusions (see Figure 3 and Supplementary Figure S3). Experimental details and data processing were as described for Table 1. At least three independent experiments were performed with similar outcome for each PH domain.

SUPPLEMENTARY FIGURES

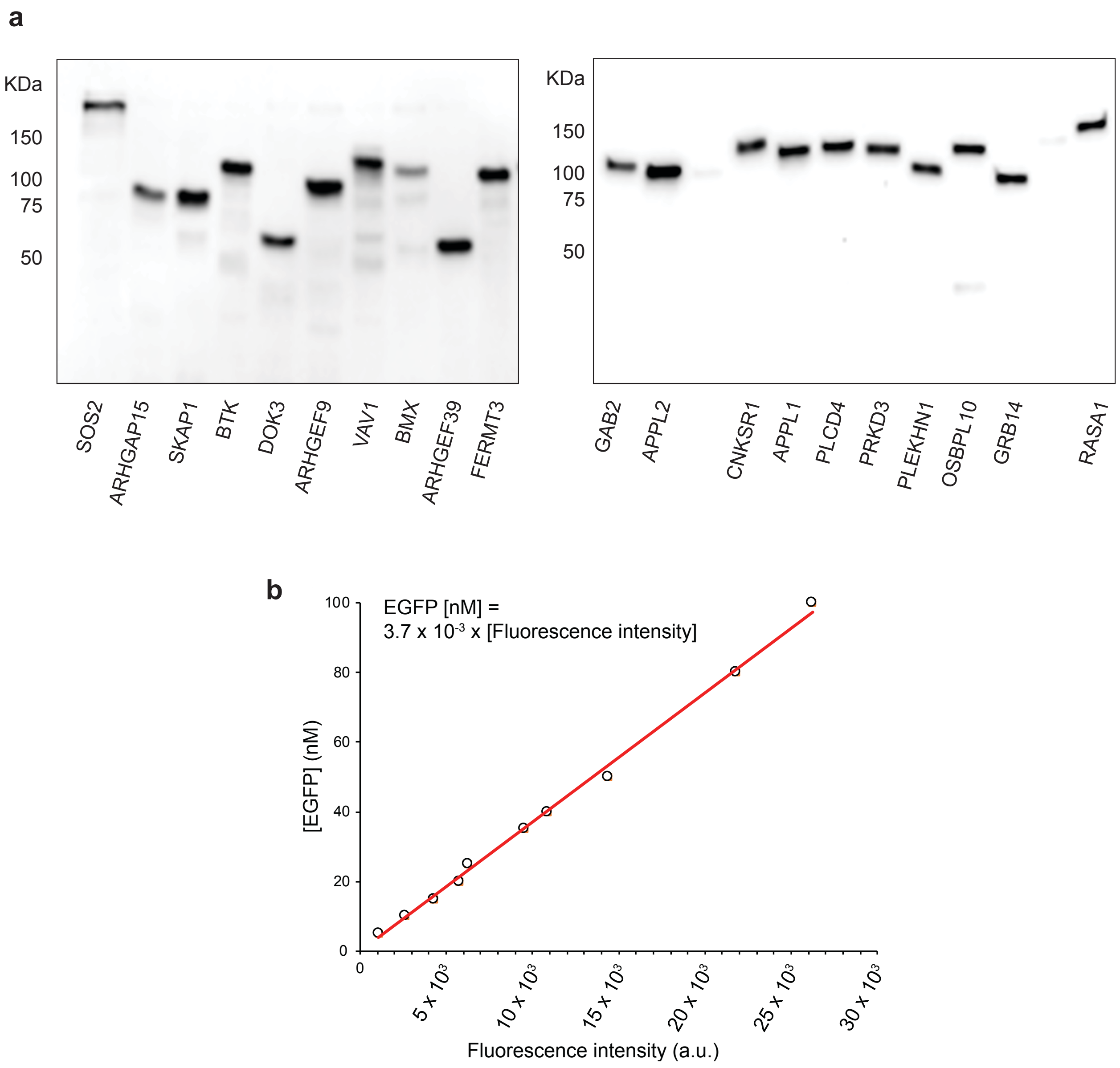

Supplementary Figure 1. Characterization of EGFP-fusion proteins for lipid-SiMPull assay.

(a) Examples of EGFP-fusion proteins transiently expressed in HEK293 cells. Cell lysates were subjected to western blotting with an anti-GFP antibody. Each fusion protein was analyzed for expression in at least 3 independent experiments. **(b)** Fluorescence of pure recombinant EGFP (ex 488 / em 520) was measured at 10 different concentrations on a Spectramax GeminiXPS plate reader (Molecular Devices) to yield a linear standard curve. EGFP [nM] = 3.7×10^{-3} x [Fluorescence Intensity(a.u.)]. $R^2 = 0.9969$. Source data are provided as a Source Data file. The experiment was repeated 3 times with similar results.

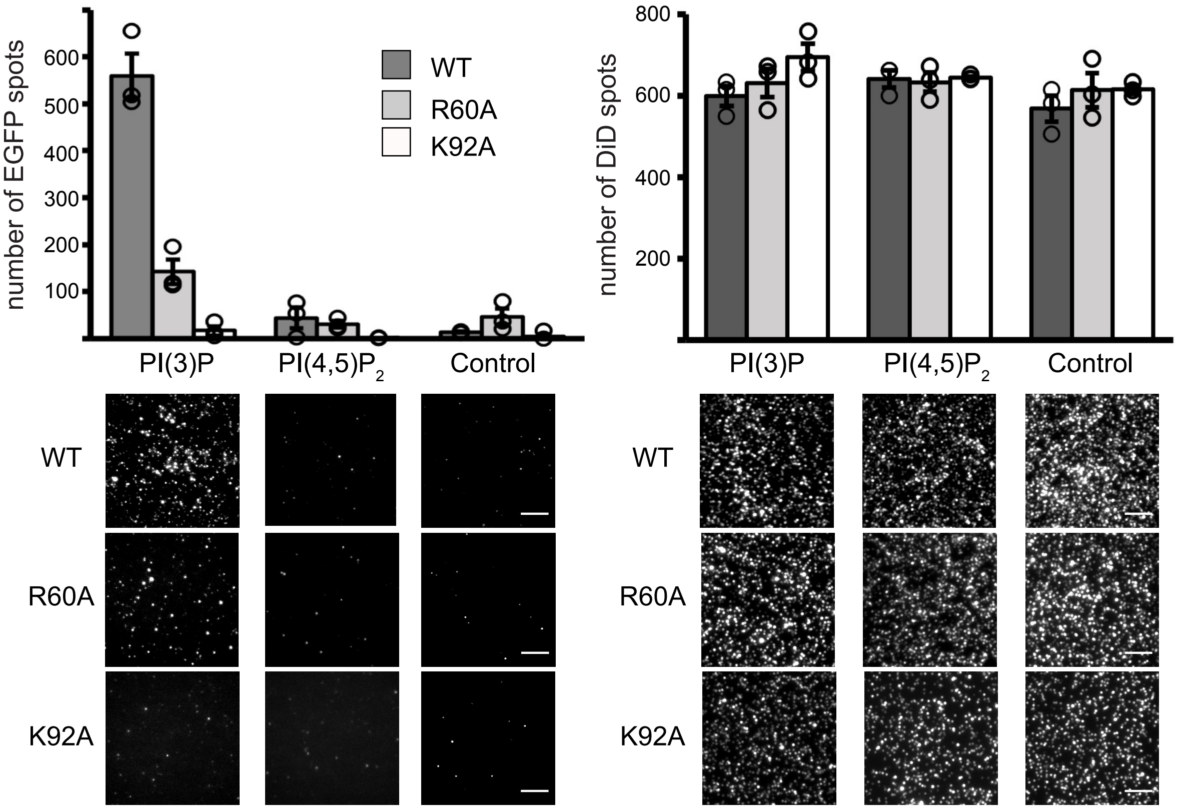

Supplementary Figure 2. Determining sensitivity of lipid-SiMPull assay.

EGFP-p40PhoxPX and mutants were expressed in HEK293 cells, and cell lysates (5 nM EGFP-fusion) were subjected to SiMPull assay with PI(3)P, PI(4,5)P_2 and PC (control) vesicles. The numbers of EGFP and DiD (vesicle) spots were quantified as described in Figure 1 legend and presented in the graphs. Representative images are shown below the graphs. Data are presented as mean ± SEM (n = 3 independent experiments). Source data are provided as a Source Data file. Scale bars: 5 μm.

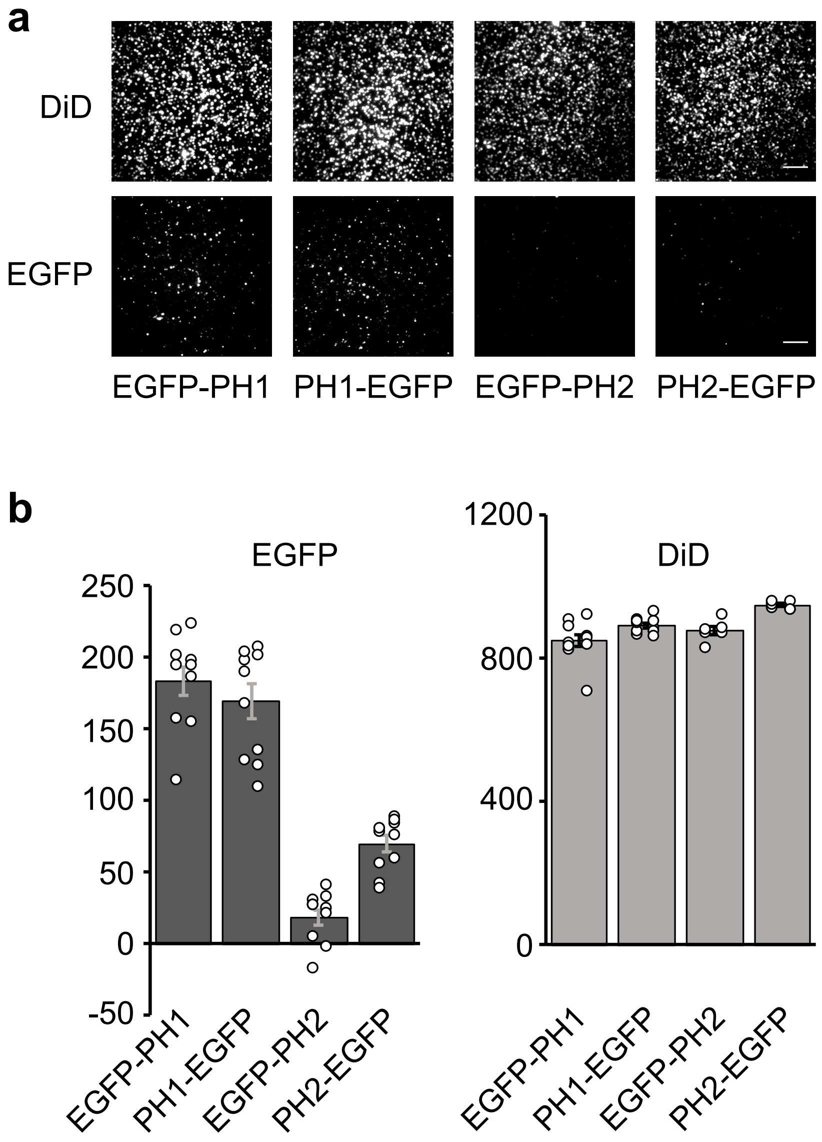

Supplementary Figure 3. Binding of ARHGEF3 PH domain to PI(4,5)P_2.

Various EGFP fusions of ARHGEF3-PH domain were expressed in HEK293 cells, and cell lysates (5 nM EGFP-fusion) were subjected to SiMPull assay with PI(4,5)P_2 vesicles. EGFP was fused at either terminus of the PH as indicated by the names of the constructs. PH1: aa320-455. PH2: aa299-466. **(a)** Representative images of EGFP and DiD. Scale bars: 5 µm. **(b)** Numbers of fluorescent molecules per image area are shown. Data are presented as mean ± SEM. $N \geq 10$ images for EGFP; $N \geq 6$ images for DiD. Source data are provided as a Source Data file, which lists the exact N number for each data point. Three independent experiments were performed with similar results.

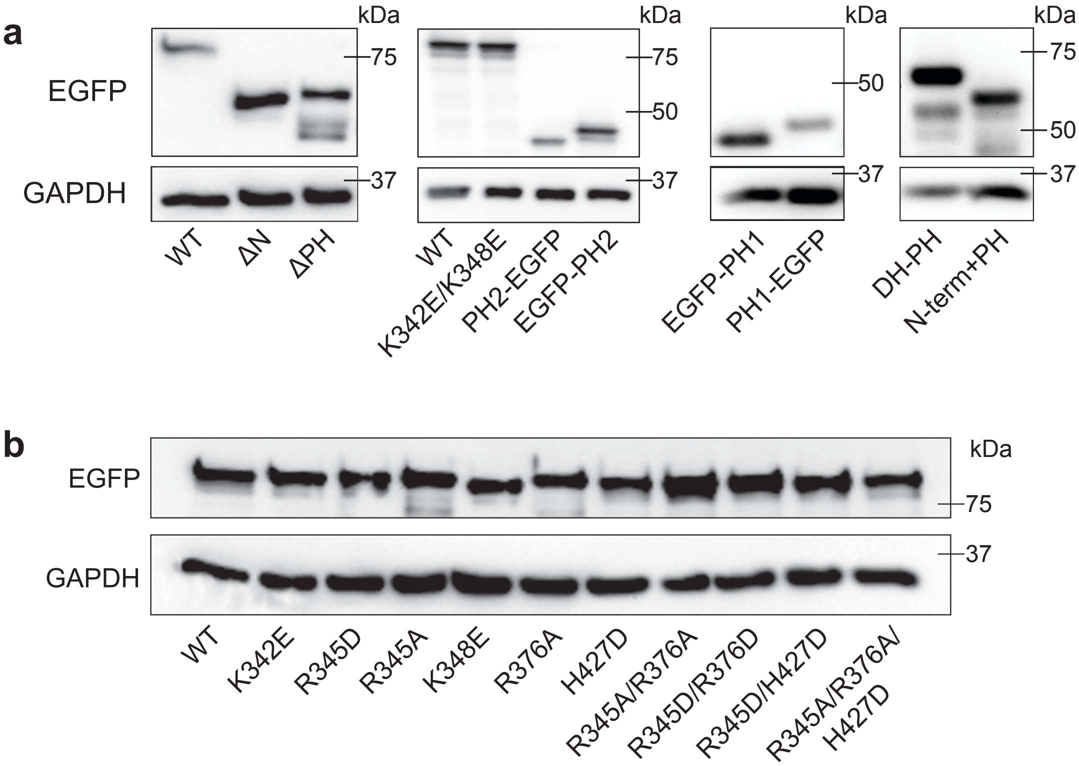

Supplementary Figure 4. Expression of ARHGEF3 truncations and mutants as EGFP fusion proteins.

HEK293 cells were transiently transfected to express various truncations **(a)** or point mutants **(b)** of ARHGEF3 fused to EGFP. Cell lysates were analyzed by western blotting with an anti-GFP antibody, and anti-GAPDH blotting served as loading control. EGFP was tagged at either terminus of the PH domain (EGFP-PH or PH-EGFP). All other constructs have the EGFP at their N-terminus. PH1: aa320-455. PH2: aa299-466. DH-PH: aa125-455. N-term+PH: aa1-125+aa320-455. ΔN: aa118-526. ΔPH: aa304-466 deleted. Uncropped and unprocessed western blot images are provided as a Source Data file. Each recombinant protein was analyzed for expression in at least 2 independent experiments.

a

PROTEIN	LIPIDS BOUND WITH MAMMALIAN LYSATES	LIPIDS BOUND WITH BACTERIAL LYSATES
ARHGEF3	$PI(4,5)P_2$, $PI(3,5)P_2$	$PI(4,5)P_2$
ARHGEF16	PI(3)P	PI(3)P
DEF6	$PI(3,4)P_2$	$PI(3,4,5)P_3$, $PI(3,4)P_2$
ITK	$PI(3,4,5)P_3$	$PI(3,4,5)P_3$

b

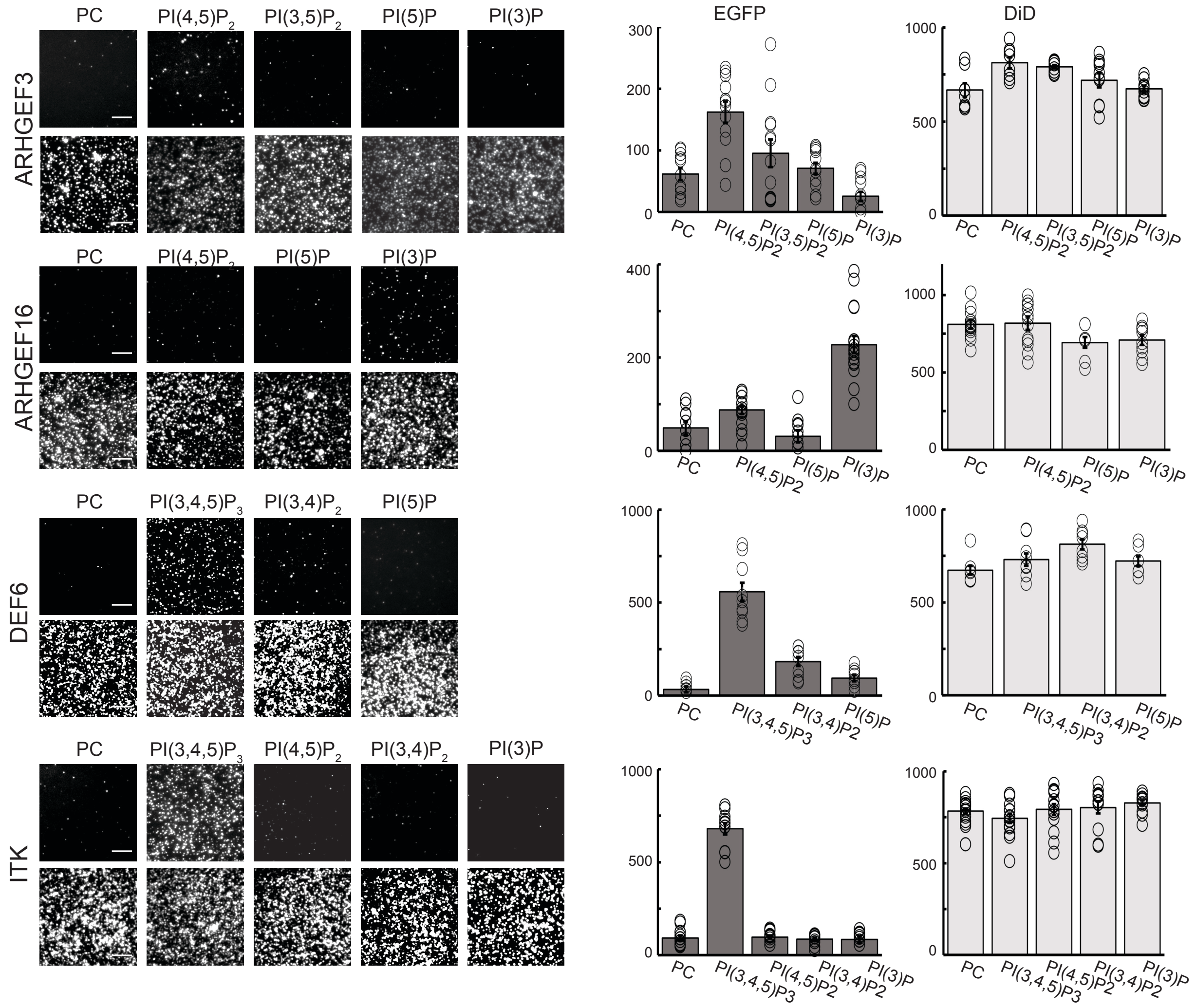

Supplementary Figure 5. Results of lipid SiMPull assays with bacterial lysates.

Bacterial lysates expressing SUMO-EGFP-fusion proteins (5 nM EGFP-fusion) were subjected to SiMPull assays. **(a)** The PH domain-containing proteins assayed and their PIP binding profiles in mammalian versus bacterial cell lysates. **(b)** Representative images of assays are shown. Scale bars: 5 µm. Numbers of fluorescent molecules per image area are shown at the right. Data are presented as mean ± SEM. N ≥ 10 images for EGFP; N ≥ 6 images for DiD. Source data are provided as a Source Data file, which lists the exact N number for each data point. Three independent experiments were performed with similar results.

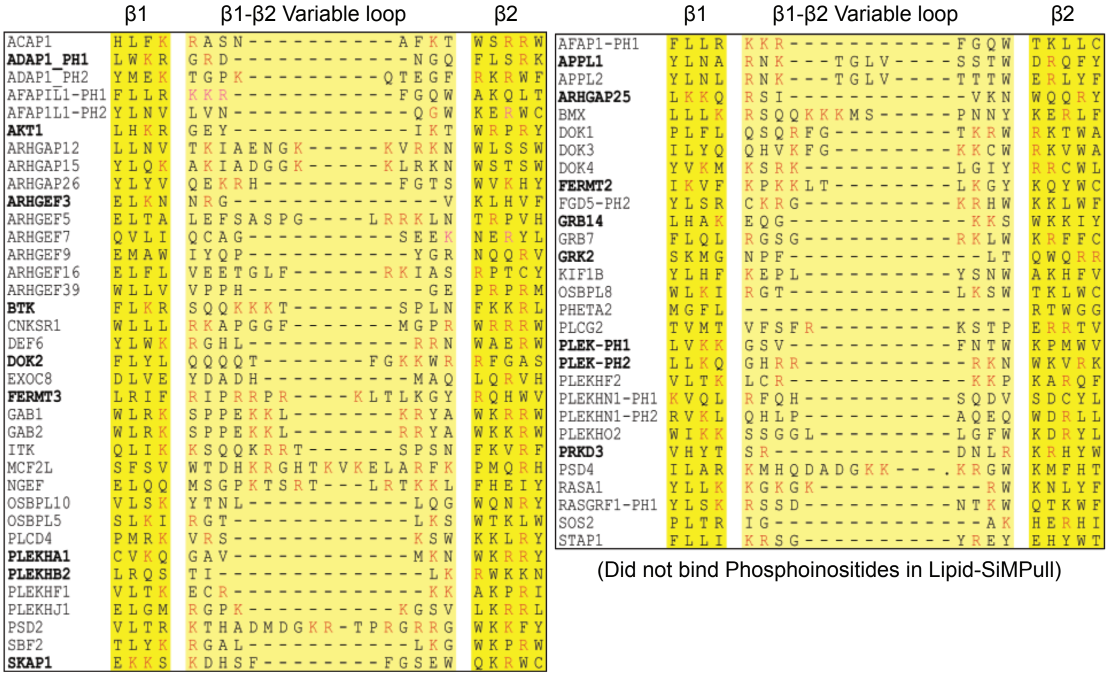

Supplementary Figure 6. A reported sequence motif is present in the PH domains of both PIP-binding and non-binding PH domain-containing proteins.

Sequence alignment of PIP-binding (left) and non-binding (right) PH domains in the β1- β2 region. Bold: PH domains with reported crystal structures. Red: residues conforming to the $KX_n(K/R)XR$ motif. Three PH domains (AFAP1-PH2, FGD5-PH1 and RASGRF1-PH2) were not shown in the alignment due to an unusually short β1 sheet.

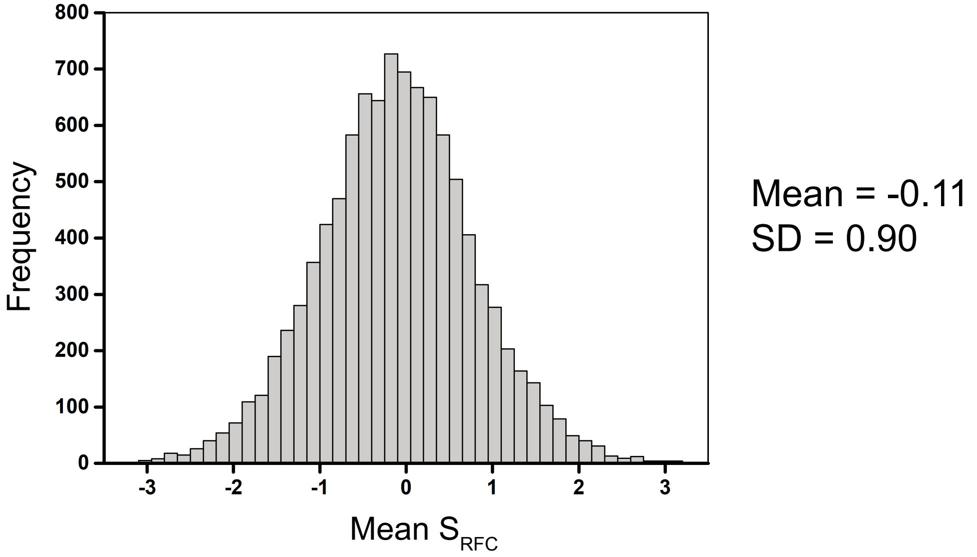

Supplementary Figure 7. Determining a cut-off S_{RFC} score for PIP-binding prediction.

The sequences of 242 human PH domains were scrambled 10,000 times, and the S_{RFC} for each scrambled sequence was calculated. The distribution of mean S_{RFC} is shown, with the overall mean and standard deviation indicated. Source data are provided as a Source Data file.
