# Supplementary material for: Redefining the specificity of phosphoinositide-binding by human PH domain-containing proteins
Source: Nat Commun. 2021 Jul 15;12:4339. doi: 10.1038/s41467-021-24639-y (PMC8282632; doi:10.1038/s41467-021-24639-y)
Supplement: Supplementary file 3 — Description of Additional Supplementary Files [file 41467_2021_24639_MOESM3_ESM.docx]

**Description of Additional Supplementary Files**

**File Name:** Supplementary Data 1

**Description:** Lipid-SiMPull assay data for 67 human PH domain-containing proteins. The results of three independent experiments are shown as EGFP spots after subtracting background. The vesicles contained 5% PIP. Ten proteins were also assayed with vesicles containing 20% PIP. Results above the threshold of binding (100) are highlighted.

**File Name:** Supplementary Data 2

**Description:** Sequence alignment and SRFC scores of 67 PH domains investigated by SiMPull.

**File Name:** Supplementary Data 3

**Description:** Sequence alignment and SRFC scores of 175 human PH domains.

**File Name:** Supplementary Data 4

**Description:** Lipid-SiMPull assay data for validation of RFC prediction of PIP binding. Human PH-domain containing proteins predicted to bind (20, top list) or do not bind PIPs (11, bottom list) were assayed against PIP vesicles shown. The number of EGFP spots after subtracting background for each assay is listed. The results of three independent experiments are shown. Results above the threshold of binding (100) are highlighted.
