# Supplementary material for: Redefining the specificity of phosphoinositide-binding by human PH domain-containing proteins
Source: Nat Commun. 2021 Jul 15;12:4339. doi: 10.1038/s41467-021-24639-y (PMC8282632; doi:10.1038/s41467-021-24639-y)
Supplement: Supplementary file 8 — Reporting Summary [file 41467_2021_24639_MOESM8_ESM.pdf]

nature research

Corresponding author(s): Jie Chen
Last updated by author(s): Jun 2, 2021

Reporting Summary

Nature Research wishes to improve the reproducibility of the work that we publish. This form provides structure for consistency and transparency in reporting. For further information on Nature Research policies, see our Editorial Policies and the Editorial Policy Checklist.

Statistics

For all statistical analyses, confirm that the following items are present in the figure legend, table legend, main text, or Methods section.

n/a	Confirmed	
☐	☒	The exact sample size (n) for each experimental group/condition, given as a discrete number and unit of measurement
☐	☒	A statement on whether measurements were taken from distinct samples or whether the same sample was measured repeatedly
☐	☒	The statistical test(s) used AND whether they are one- or two-sided<br>*Only common tests should be described solely by name; describe more complex techniques in the Methods section.*
☒	☐	A description of all covariates tested
☒	☐	A description of any assumptions or corrections, such as tests of normality and adjustment for multiple comparisons
☐	☒	A full description of the statistical parameters including central tendency (e.g. means) or other basic estimates (e.g. regression coefficient) AND variation (e.g. standard deviation) or associated estimates of uncertainty (e.g. confidence intervals)
☐	☒	For null hypothesis testing, the test statistic (e.g. F, t, r) with confidence intervals, effect sizes, degrees of freedom and P value noted<br>*Give P values as exact values whenever suitable.*
☒	☐	For Bayesian analysis, information on the choice of priors and Markov chain Monte Carlo settings
☒	☐	For hierarchical and complex designs, identification of the appropriate level for tests and full reporting of outcomes
☒	☐	Estimates of effect sizes (e.g. Cohen's d, Pearson's r), indicating how they were calculated

Our web collection on statistics for biologists contains articles on many of the points above.

Software and code

Policy information about availability of computer code

Data collection	The smFRET package was used to acquire images for TIRF microscopy, which is developed and maintained by Dr. T J Ha laboratory and is available from Github: https://github.com/Ha-SingleMoleculeLab
Data analysis	Microsoft Excel 2016, Origin 9.7, Fiji(ImageJ2), IDL 6.2SE, MATLAB R2016a, PROMALS3D (no version number from the developer), Patch Finder Plus 2.3, Pymol 2.5, smFRET(2014)

For manuscripts utilizing custom algorithms or software that are central to the research but not yet described in published literature, software must be made available to editors and reviewers. We strongly encourage code deposition in a community repository (e.g. GitHub). See the Nature Research guidelines for submitting code & software for further information.

Data

Policy information about availability of data

All manuscripts must include a data availability statement. This statement should provide the following information, where applicable:
- Accession codes, unique identifiers, or web links for publicly available datasets
- A list of figures that have associated raw data
- A description of any restrictions on data availability

The Data Availability statement is included in the manuscript as follows:
All data generated or analyzed during this study are included in the manuscript, its supplementary information, and source data file, and from the corresponding author upon reasonable request. Source data underlying all figures, and the original western blotting images (iBright, Invitrogen) are provided with this manuscript as source data files.

Field-specific reporting

Please select the one below that is the best fit for your research. If you are not sure, read the appropriate sections before making your selection.

- [x] Life sciences
- [ ] Behavioural & social sciences
- [ ] Ecological, evolutionary & environmental sciences

For a reference copy of the document with all sections, see nature.com/documents/nr-reporting-summary-flat.pdf

Life sciences study design

All studies must disclose on these points even when the disclosure is negative.

Sample size	This study entails in vitro (cell culture) experiments exclusively. No statistical methods were used to predetermine sample sizes. The vast majority of the experiments were performed a minimum of 3 times with independent samples. The sample size was chosen based on previously published similar work and commonly adopted standards in the field.
Data exclusions	Each batch of the lipid vesicles was tested against a known binding partner, and if it failed to bind this protein we did not proceed with the experiment using this batch of vesicles. Sometimes this positive control experiment was performed at the same time as the experiment, which led to exclusion of those data when the positive control failed using the same batch of vesicles.
Replication	As described above, at least 3 independent experiments were performed for each type of experiments. When the positive and negative controls were validated, all the experiments reported in this manuscript were reproducible.
Randomization	The cells in the experiments were of different passages and various densities, and they were randomly assigned to the transfection of different plasmids and different repeats of transfection of the same plasmids.
Blinding	Whereas blinding was not used in data analysis in this study, the vast majority of the experiments were performed without a predicted outcome and by at least two different researchers in parallel without prior knowledge of each other's results. For single-molecule spot counting we used computer algorithm based on point spread function for all images, and it did not involve any manual counting.

Reporting for specific materials, systems and methods

We require information from authors about some types of materials, experimental systems and methods used in many studies. Here, indicate whether each material, system or method listed is relevant to your study. If you are not sure if a list item applies to your research, read the appropriate section before selecting a response.

Materials & experimental systems

n/a	Involved in the study	
☐	☒	Antibodies
☐	☒	Eukaryotic cell lines
☒	☐	Palaeontology and archaeology
☒	☐	Animals and other organisms
☒	☐	Human research participants
☒	☐	Clinical data
☒	☐	Dual use research of concern

Methods

n/a	Involved in the study	
☒	☐	ChIP-seq
☒	☐	Flow cytometry
☒	☐	MRI-based neuroimaging

Antibodies

Antibodies used	Anti-GST (B-14) antibody (#sc-138) was obtained from Santa Cruz Biotechnology (Dilution 1:2000). Anti-GFP (# 600-106-215) was from Rockland Immunochemicals, Inc (Dilution 1:2000). The following antibodies were obtained from Cell Signaling Technology: pSer473-Akt (9271) (Dilution 1:1000), Akt (9272) (Dilution 1:2000), RhoA (2117)(1:1000), and GAPDH (2118)(1:2000). Peroxidase-conjugated anti-rabbit IgG (115-036-003) (dilution 1:1000) and anti-mouse IgG (111-036-003)(dilution 1:1000) were obtained from Jackson Immuno Research Laboratories. HRP Rabbit anti-Goat IgG (H+L), Invitrogen #A27014, (dilution 1:2500).
Validation	Some of the primary antibodies were validated in our own lab for specificity, using cell lysates with and without the target protein. GFP and GST antibodies were validated using recombinant proteins with and without the tag. Akt antibody was validated using cell lysates expressing a tagged recombinant protein that has a different mobility than the endogenous Akt. Phospho-Akt antibody was validated with lysates of HEK293 cells starved and stimulated with insulin. GAPDH and RhoA antibodies were validated by the manfacturer (Cell Signaling Technology).

Eukaryotic cell lines

Policy information about cell lines

Cell line source(s)	HEK293 and NIH3T3 were both obtained from ATCC originally.
Authentication	Cell lines used in this study have not been authenticated commercially since arrival at our lab. However, cell morphology and signaling response to distinct stimuli are monitored regularly in our lab to assure cell line identity.
Mycoplasma contamination	All cell lines are tested for mycoplasma contamination by PCR-based assays on a bi-monthly basis in our lab. The HEK293 cells were tested positive for mycoplasma and cultured in a separate incubator from all other cell lines. All other cell lines in our lab are mycoplasma-negative.
Commonly misidentified lines (See ICLAC register)	This study did not use any commonly misidentified cell line.
