# Supplementary material for: Redefining the specificity of phosphoinositide-binding by human PH domain-containing proteins
Source: Nat Commun. 2021 Jul 15;12:4339. doi: 10.1038/s41467-021-24639-y (PMC8282632; doi:10.1038/s41467-021-24639-y)

Figure 5b

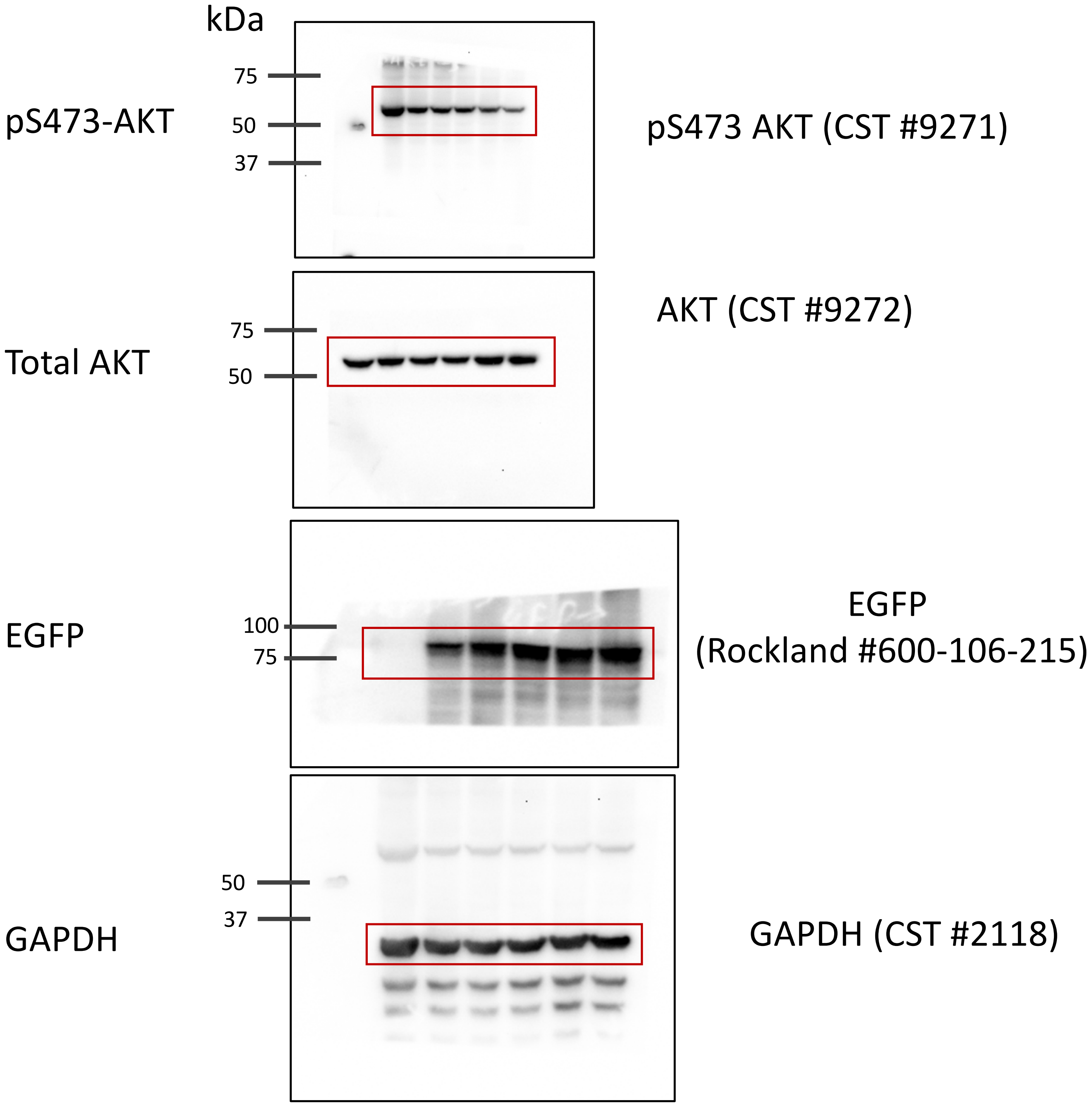
kDa
pS473-AKT
75
50
37
pS473 AKT (CST #9271)
Total AKT
75
50
AKT (CST #9272)
EGFP
100
75
EGFP
(Rockland #600-106-215)
GAPDH
50
37
GAPDH (CST #2118)

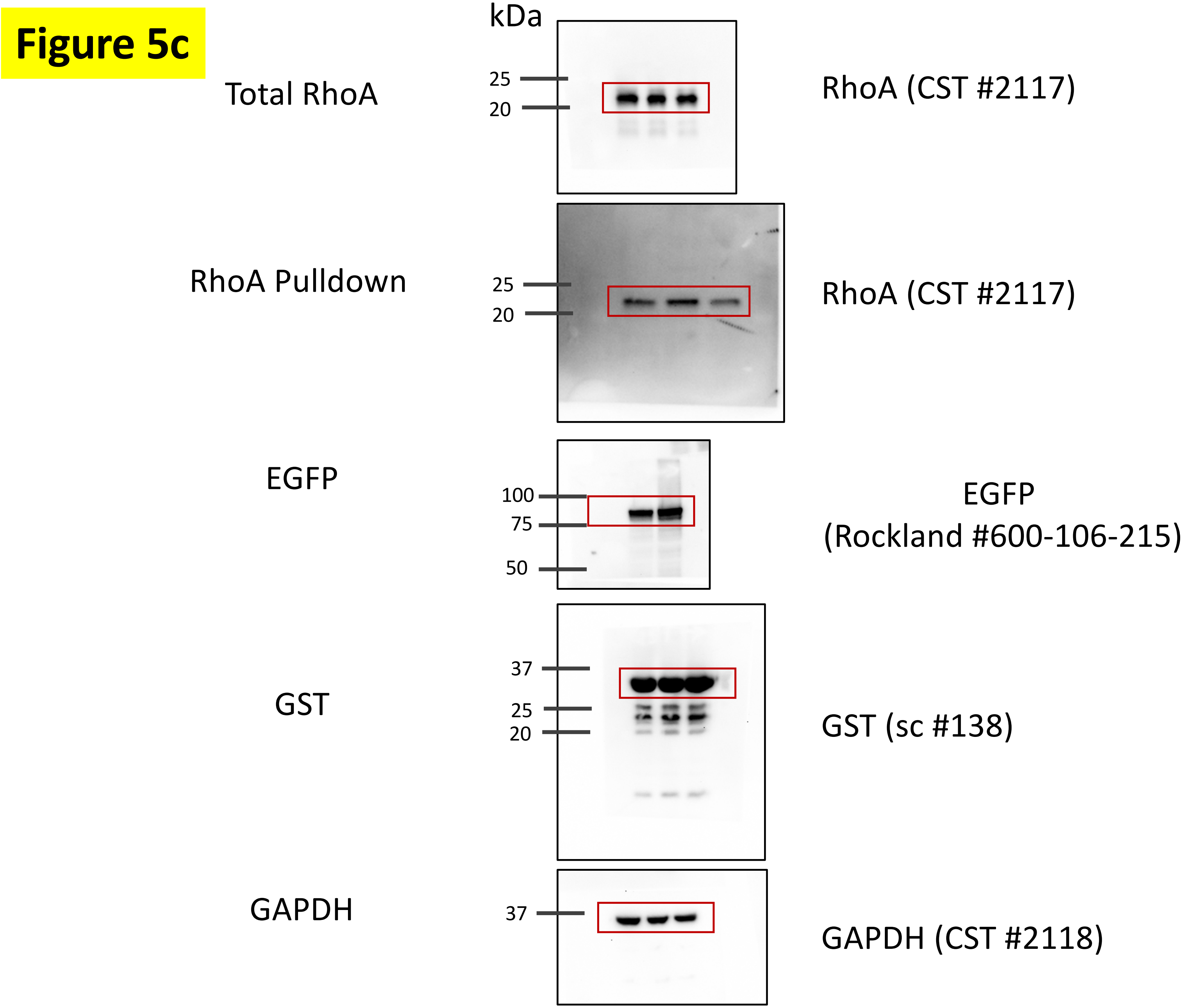
Figure 5c
kDa
Total RhoA
25
20
RhoA (CST #2117)
RhoA Pulldown
25
20
RhoA (CST #2117)
EGFP
100
75
50
EGFP
(Rockland #600-106-215)
GST
37
25
20
GST (sc #138)
GAPDH
37
GAPDH (CST #2118)

## Supplementary Figure 1a

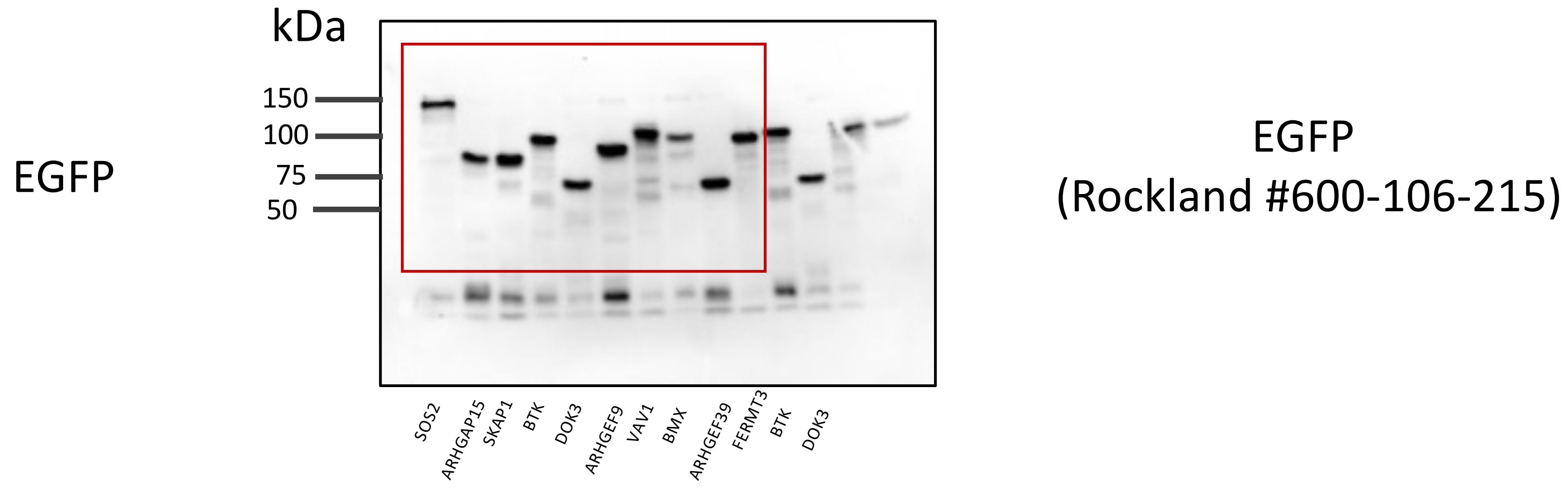


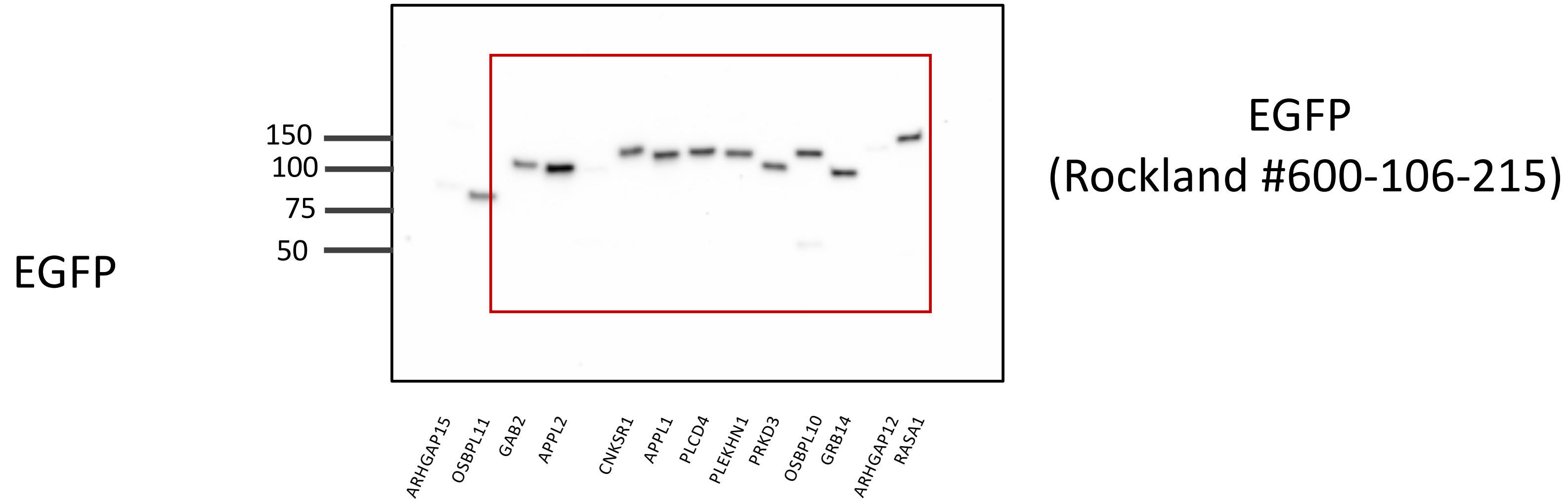

**Supplementary Figure 4a**

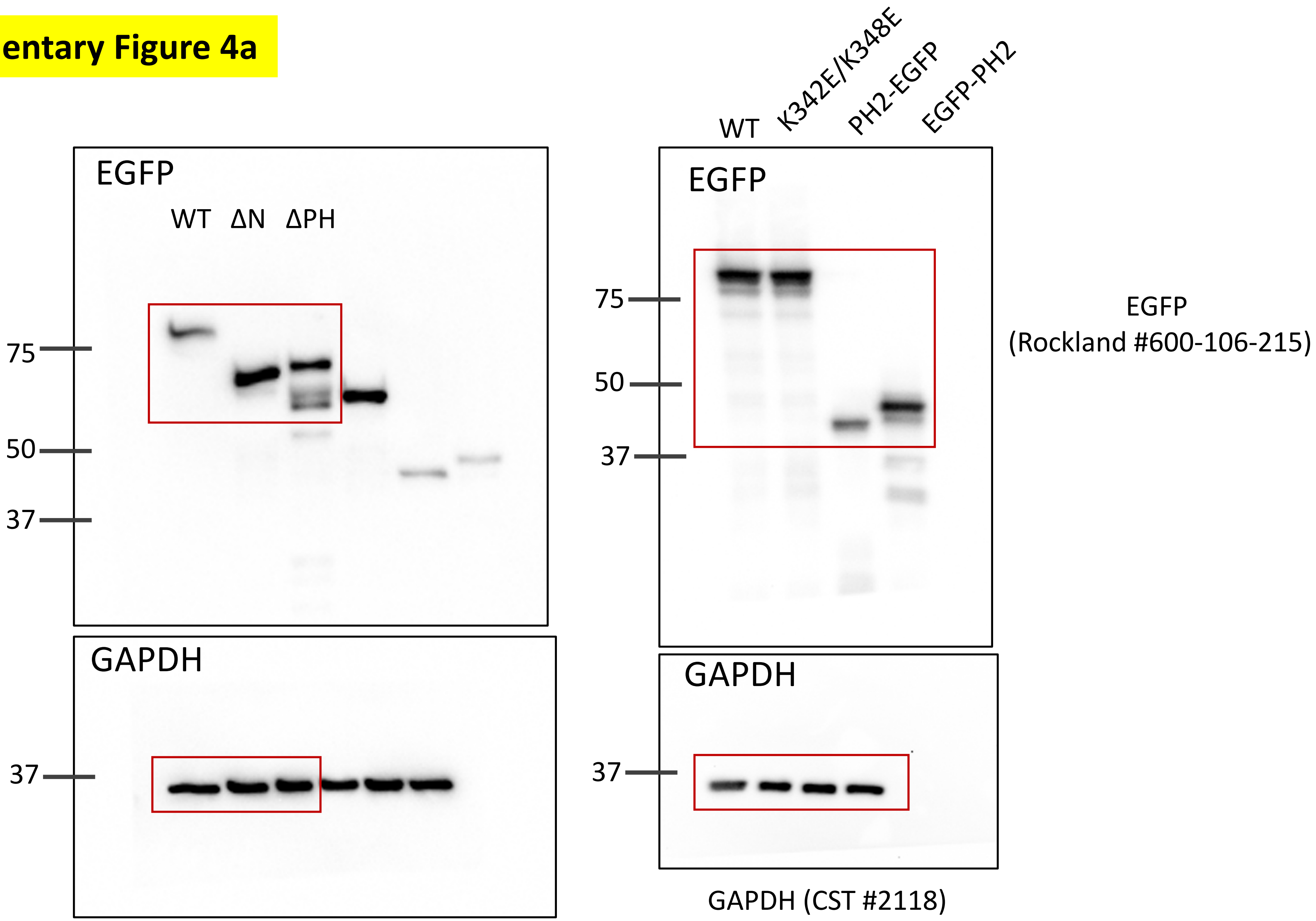


Different constructs of ARHGEF3 tagged with EGFP

**Supplementary Figure 4a**

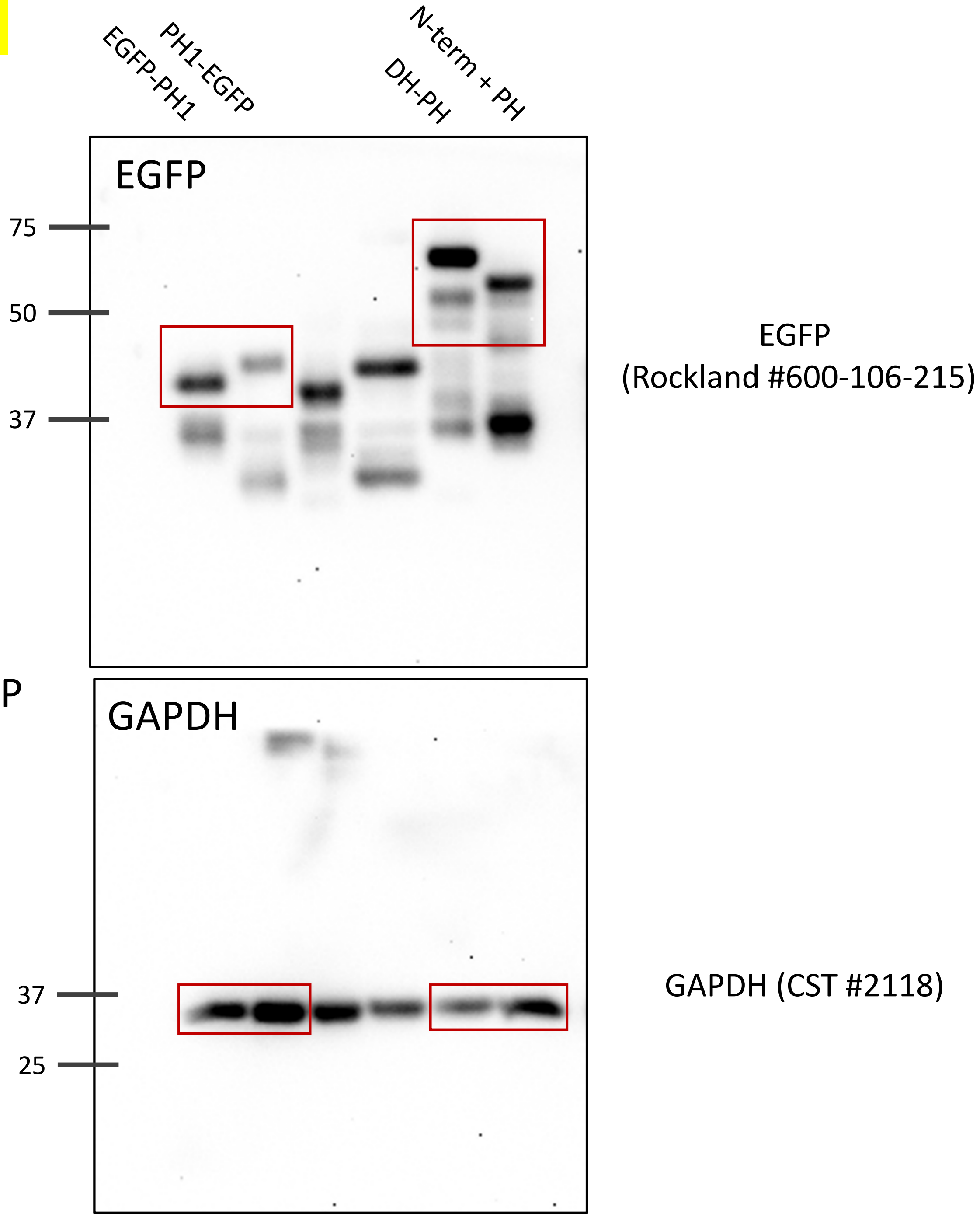


Different constructs of ARHGEF3 tagged with EGFP

**Supplementary Figure 4b**

Different mutants of ARHGEF3 tagged with EGFP

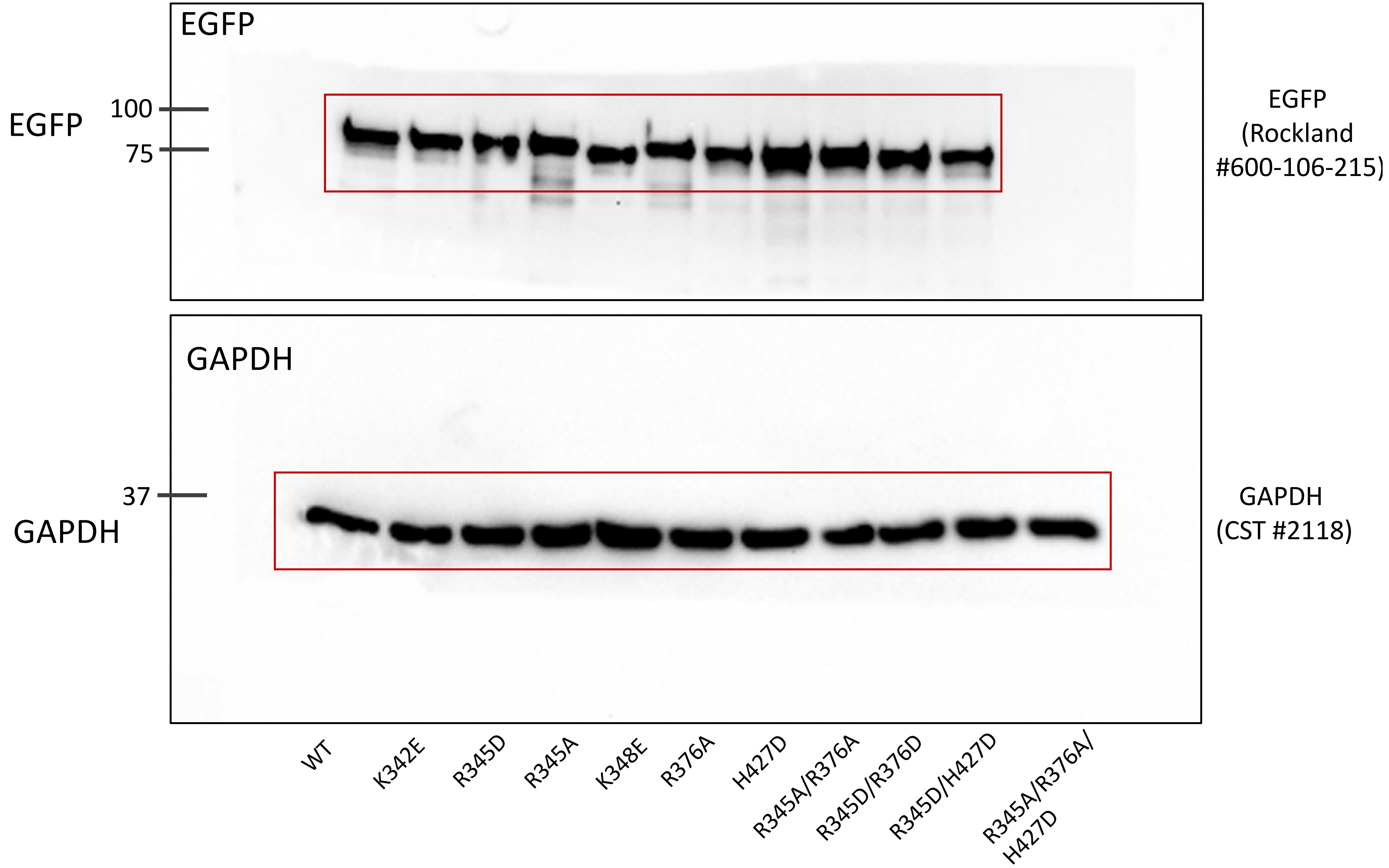

Supplement: Supplementary file 9 — Source Data [file 41467_2021_24639_MOESM9_ESM.zip › Source_Data_uncropped blots_rev.pdf]
